# Proteome trait regulation of marine *Synechococcus* elemental stoichiometry under global change

**DOI:** 10.1093/ismejo/wrae046

**Published:** 2024-03-21

**Authors:** Nathan S Garcia, Mingyu Du, Michele Guindani, Matthew R McIlvin, Dawn M Moran, Mak A Saito, Adam C Martiny

**Affiliations:** Department of Earth System Science, University of California, Irvine, Irvine, CA 92697, United States; Department of Statistics, University of California, Irvine, Irvine, CA 92697, United States; Department of Biostatistics, University of California, Los Angeles, Los Angeles, CA 90095, United States; Marine Chemistry and Geochemistry Department, Woods Hole Oceanographic Institution, Woods Hole, MA 02543, United States; Marine Chemistry and Geochemistry Department, Woods Hole Oceanographic Institution, Woods Hole, MA 02543, United States; Marine Chemistry and Geochemistry Department, Woods Hole Oceanographic Institution, Woods Hole, MA 02543, United States; Department of Earth System Science, University of California, Irvine, Irvine, CA 92697, United States; Department of Ecology and Evolutionary Biology, University of California, Irvine, Irvine, CA 92697, United States

**Keywords:** Synechococcus, nutrient stress, temperature stress, resource allocation, elemental stoichiometry, proteome, traits, growth rate hypothesis, global change

## Abstract

Recent studies have demonstrated regional differences in marine ecosystem C:N:P with implications for carbon and nutrient cycles. Due to strong co-variance, temperature and nutrient stress explain variability in C:N:P equally well. A reductionistic approach can link changes in individual environmental drivers with changes in biochemical traits and cell C:N:P. Thus, we quantified effects of temperature and nutrient stress on *Synechococcus* chemistry using laboratory chemostats, chemical analyses, and data-independent acquisition mass spectrometry proteomics. Nutrient supply accounted for most *C:N:P_cell_* variability and induced tradeoffs between nutrient acquisition and ribosomal proteins. High temperature prompted heat-shock, whereas thermal effects via the “translation-compensation hypothesis” were only seen under P-stress. A Nonparametric Bayesian Local Clustering algorithm suggested that changes in lipopolysaccharides, peptidoglycans, and C-rich compatible solutes may also contribute to C:N:P regulation. Physiological responses match field-based trends in ecosystem stoichiometry and suggest a hierarchical environmental regulation of current and future ocean C:N:P.

## Introduction

The relative composition of elements in phytoplankton (i.e. C:N:P) is central to ocean functioning. This includes environmental interactions with biodiversity [1], ecological and trophic exchanges [2, 3], nitrogen fixation [4], and the biological pump [5]. The C:N:P composition of ocean phytoplankton has been assumed constant for many decades—i.e. the Redfield Ratio of 106:16:1 [6]. Although field studies now demonstrate strong regional and temporal variation in elemental stoichiometry of marine communities [7–9], the underlying controls of ocean C:N:P are not well-constrained. A trait-based approach can provide a mechanistic biochemical understanding of C:N:P regulation and improve modeled ecosystem responses to global change.

Several biochemical mechanisms are thought to control phytoplankton elemental ratios [10, 11], but are difficult to identify due to multiple influential factors. The most prominent hypotheses involve element storage and regulation of P-rich ribosomes—the machinery for biosynthesis. The nutrient supply theory posits that cells are frugal under nutrient scarcity but increase storage when nutrients are abundant [12–16]. This mechanism can result in a correspondence between nutrient concentrations and *C:N:P_cell_* [5]. The translation compensation hypothesis posits that P-rich ribosomes are abundant at low temperature to compensate for slow translational activity, leading to depressed C:P and N:P ratios in high-latitude ecosystems [17, 18]. Finally, the growth rate hypothesis posits that cellular growth also has specific requirements for ribosomes that can directly affect *C:N:P_cell_*, resulting in tradeoffs with other cell components [19, 20]. The challenge is that each of these biochemical mechanisms can explain current field observations equally well due to latitudinal co-variance between nutrient stress, temperature, and growth status of phytoplankton. Controlled laboratory experiments, mimicking balanced growth conditions in the oceans, provide a way to distinguish environmental effects on specific traits and elemental allocations.

Biomolecular studies suggest that phytoplankton employ several additional mechanisms to manage environmental stress. For example, nutrient stress influences N-rich nutrient acquisition proteins (NAPs) [21] and phycobilisomes [22]. Sulfolipids can replace phospholipids in membranes of Cyanobacteria under P-stress [23–25], thereby reducing the P quota. Polyphosphates (poly-P) can store P [16], but also serve a variety of physiological functions [26, 27]. More recently, the periplasm was suggested as a nutrient docking and storage site that assists in cell nourishment [15]. However, the contribution of these molecular mechanisms to cellular elemental stoichiometry is unclear [10], particularly under balanced growth conditions, and we have a limited view of the contribution of each biochemical mechanism and associated traits to the regulation of *C:N:P* in the field.

Here, we quantified the relative impacts of temperature and nutrient stress on cellular C:N:P in one of the largest contributors to ocean primary production, *Synechococcus* [28]. To account for growth rate effects, we normalized to continuous growth with a chemostat culture design. To understand the trait-based biochemical regulation of cell quotas, we integrated analyses of cellular elemental resource allocations with data-independent acquisition mass spectrometry (DIA-MS) proteomics. Combined, these analyses provide a molecular view of trait regulation of C:N:P in an abundant marine phytoplankton.

## Materials and methods

### Experimental design and elemental analysis

We grew *Synechococcus* cultures (WH8102) in polycarbonate bottles with a continuous method used previously [29] in artificial seawater (Table S1). We used two concentration ratios of macronutrients (NO_3_^−^:PO_4_^3−^ = 1.7 and 80) and three levels of temperature (20, 24, and 28°C) with a slow dilution rate to ensure treatment-wise culture stability. White light was supplied at 125 μmol quanta m^−2^ s^−1^ on a 12-h:12-h light:dark cycle. Equilibria were monitored by measuring culture cell density and forward scatter (*FSC_H_*) with a Novocyte flow cytometer 1000 (Acea Biosciences, Inc., San Diego, CA). Biomass was collected after an acclimation period on Days 38, 43, 47, 50, and 57 for particulate organic matter, nutrient analysis, cellular proteins, culture cell density, and *FSC_H_* (Fig. S1). Particulate organic carbon and nitrogen (150 ml) and phosphorus (50 ml) were collected at the midpoint of the light period with glass fiber filters (Whatman, GE Healthcare, Little Chalfont, Buckinghamshire, UK) and measured using a Flash EA1112 gas chromatograph (Thermo Scientific) and a Genesys 10S UV–vis spectrophotometer (Thermo Scientific, Madison, WI) at 885 nm following methods described by Michaels *et al*. [30]. Culture cell density and *FSC_H_* were measured in samples collected for biomass. Cells for proteome analysis were collected with a 47 mm polycarbonate filter (0.2 μm pore size) 7–8 h into the light period, pelleted by centrifugation (21 130 *g* for 3 min), flash frozen in liquid nitrogen, and stored at −80°C.

### Protein extraction and peptide preparation

Proteins were extracted by heating pelleted cells at 95°C for 10 min and gently shaking at room temperature for 30 min in a buffer solution (400 μl–1760 μl; 50 mM N-(2-Hydroxyethyl)piperazine-N′-(2-ethanesulfonic acid (HEPES) pH 8.5 [Boston BioProducts #BB-2082], 1% sodium dodecyl sulfate (SDS) in high-performance liquid chromatography (HPLC) grade water) before centrifuging at 14 100 *g* for 20 min at room temperature and removing the supernatant. Sodium dodecyl sulfate (1%) is a strong detergent for diverse matrices including cell membranes [31]. Benzonase nuclease (50 units; Novagen #70746–3) was added to 400 μl extracted protein sample and incubated at 37°C for 30 min. Samples were reduced by adding 20 μl of 200 mM dithiothreitol (DTT) (Fisher #BP172–5) in 50 mM HEPES pH 8.5 at 45°C for 30 min and alkylated with 40 μl of 400 mM iodoacetamide (Acros #122270050) in HEPES pH 8.5 for 30 min at 24°C. The reaction was quenched by adding 40 μl of 200 mM DTT in 50 mM HEPES pH 8.5. SpeedBead Magnetic Carboxylate Modified Particles (GE Healthcare #65152105050250 and #45152105050250) were prepared according to Hughes *et al.*[31] and added (20 μg/μl) to 400 μl of extracted protein sample. Samples were incubated with formic acid (pH of 2–3) and washed with ethanol and acetonitrile using a magnetic rack. Protein was measured with the bicinchoninic acid (BCA) method (Thermo Scientific Micro BCA Protein Assay Kit #23235) and digested overnight at 37°C with 1 part trypsin (Promega #V5280; dissolved in HEPES pH 8.0, 0.5 μg/μl) and 25 parts protein. Peptides were washed with acetonitrile and ethanol using a magnetic rack and diluted to a target concentration of 0.1% trifluoroacetic acid or 1% formic acid and a final concentration of 1 μg/μl.

### Mass spectrometry of peptides

Similar to other analyses [32], peptides were analyzed using a Michrom Advance HPLC system coupled to a Q-Exactive mass spectrometer (Thermo Scientific instrument version 2.8) with a Michrom Advance CaptiveSpray source, using the constant injection concentration of 1 μg/μl to allow uniformity across the dataset. Samples were concentrated onto a C18 column (Reprosil-Gold, Dr Maisch GmbH) and eluted in a non-linear, 200-min gradient of formic acid and acetonitrile buffers. Full MS1 scans were performed (35 000 resolution, 3e6 AGC target, 60 ms maximum IT, 385 to 1015 m/z) with overlapping DIA scans (17 500 resolution, 1e6 AGC target, 60 ms maximum IT, 24.0 m/z isolation windows, normalized collision energy of 27, loop count 25, see Supplementary Material for expanded methods).

### Proteomic data analysis

DIA-MS sample data were analyzed using Scaffold DIA (2.2.1), converted to mzML format (ProteoWizard 3.0.11748), and individually searched against Syn8102_uniprot-proteome_UP000001422.fasta with a peptide and fragment mass tolerance of 10.0 ppm. Percolator (3.01) filtered peptides for a maximum false discovery rate of 0.01. Charged peptides (2–3) with length (6–30) were considered. EncyclopeDIA (0.9.6) selected the five highest quality fragment ions for quantitation [32]. Within the total proteome, 1231 proteins were identified with two or more representative peptides. However, we only included 1146 proteins in the broader analysis because some of the proteins were not detected across the entire sample set. Thus, we removed proteins that returned a “missing value” in three or more of the samples (10% or more), keeping only those returning two or less missing values across the 30-sample set. Mean total peptide peak areas were normalized across all samples with the Scaffold DIA Proteome Software to allow intercomparisons across samples (Proteome Software, Inc., Portland, OR; Fig. S2). We summed peak areas (*PA*s) of peptides assigned to all 1231 proteins to obtain *PA_Total_* within a given sample. We then calculated the sum of *PA*s of proteins within specific groups related to phycobilisomes, N- and P-acquisition, biosynthesis, heat shock, cell motility, photic electron transport, oxidative stress, cell structure, metals transport, and CO_2_ fixation (identifying references [33–36], see Table S7 for protein group identification), within a given sample. We compared *PA* of a protein or protein group to the *PA_Total_* for each sample (*n* = 5 for each treatment) and report model statistics for a protein or protein group % *PA_Total_*.


$$ \%\ {PA}_{Total}= protein\ or\ protein\ group\ PA/{PA}_{Total}\times 100 $$

We interpret the summed *PA* of tryptic peptides as reflective of cellular resources being deployed for each function, rather than of copy number because proteins have lengths and numbers of peptides. Moreover, although there are differences in ionization efficiency between peptides, the summed *PA* provided an aggregate metric to consider allocation of cellular resources within protein groups. Assumptions within data preparation had a very minor impact on results: the use of a more stringent two peptides per protein caused a loss of only 0.34% of *PA_Total_*, and removal of proteins missing in more than two samples resulted in a loss of only 0.33% *PA_Total_* (Table S2). We also considered a group that includes 100 proteins with the highest mean *PA*, which accounted for 74 ± SE 2.5% of *PA_Total_* (Fig. S2) indicating that <10% of the observable proteins contribute to a large majority of the protein mass. The mass spectrometry proteomics data have been deposited to the ProteomeXchange Consortium via PRIDE [1] partner repository with the dataset identifier PXD043180.

### Analysis of variance and clustering analyses

We relied on the two-way analysis of variance (ANOVA) to describe differences in cellular elemental quotas and ratios and *FSC_H_* of *Synechococcus* using the *anova2* function in Matlab (The Mathworks, Inc.). To describe variability in proteins, we relied on a variety of methods including the two-way ANOVA, Benjamini–Hochberg pairwise comparisons test, permutational multivariate analysis of variance (PERMANOVA) on protein groups using the *adonis2* function from the *vegan* package in *R*, a hierarchical clustering function for protein analysis in Matlab, and a Nonparametric Bayesian Local Clustering (NoB-LoC) algorithm.

We fit the NoB-LoC algorithm to 1146 proteins [37]. This method uses the Dirichlet process mixture model with the zero-enriched Pólya urn scheme [38] and partitions proteins into sets or biclusters that have similar distributions of relative abundance within subpartitions or subclusters, regardless of mean value (e.g. low vs. high relative abundance), thereby classifying proteins based on response patterns. To reduce stringency on biclusters, the method identifies “invariant” proteins and samples that do not follow broader distribution patterns within identified subcluster distributions.

We initialized the biclustering indicator $\omega$ by removing non-clustering proteins (singletons) from hierarchical clustering and designated them as “invariant,” meaning they do not follow distribution patterns that are similar to other proteins. There are 20 variant protein sets and 1 invariant set including 10 proteins in the initialized partition of our model. Moreover, because many biological processes involve only a small subset of proteins, we set up a prior construction of $\omega$ by assuming that a protein g is invariant (${\omega}_g=0)$ with probability $\left(1-{\pi}_0\right)$, where ${\omega}_g$is the cluster membership indicator for protein g. Here we set ${\pi}_0=0.01$, which allows a small subset of proteins to be involved in a pathway. We implemented a Markov Chain Monte Carlo simulation with 35 000 iterations with 5000 burn-in iterations. To measure the uncertainty of estimation, we used a distance metric


$$ H\left(\omega, {\omega}^{LS}\right)={\sum}_{g=1}^G\ {\sum}_{g\prime =g}^G\ \left|{d}_{g,g\prime}^{\omega }-{d}_{g,g\prime}^{\omega^{LS}}\right| $$

in which, ${d}_{g,g\prime}^{\omega }=I\left({\omega}_g={\omega}_{g\prime}\right)$ is an indicator of whether the protein g and g′ are clustered together in partition $\omega$ and ${d}_{g,g\prime}^{\omega^{LS}}$ is the clustering indicator for the estimated partition ${\omega}^{LS}$. The posterior distribution of scaled distance metric for $\omega$ is reasonable with low variability around zero (Fig. S3).

We identified 8 biclusters and 317 invariant proteins with this method. To identify relative partitioning of proteins into biclusters, we ranked them by % *PA_Total_* (Fig. S3). We then used the proportional difference from the mean log *PA* of a given protein (mean calculated across all 30 samples) and arranged proteins in order based on the proportional difference from mean values to identify proteins with similar responses to nutrients and temperature stress (Table S3).

## Results

### Changes in cellular elements and cell size

To quantify molecular trait regulation of *Synechococcus* elemental composition, we used a factorial chemostat design to grow WH8102 under a range of temperature and nutrient stress levels. The two-factorial design covered P-stress (*N:P_input_* = 80:1) and N-stress (*N:P_input_* = 1.7:1) at 20°C, 24°C, and 28°C. We measured our fixed dilution rate across treatments at 0.178 ± 0.004(mean ± SD) day^−1^, which we controlled to isolate effects of nutrient and temperature stress from growth rate effects on cellular biochemical regulation. Specifically, we measured equilibrium cellular C-, N-, and P-quotas from biomass and cell counts, *FSC_H_* (cell size proxy) and cell counts using flow cytometry, and relative protein abundances using DIA-MS proteomics.

**Figure 1:**
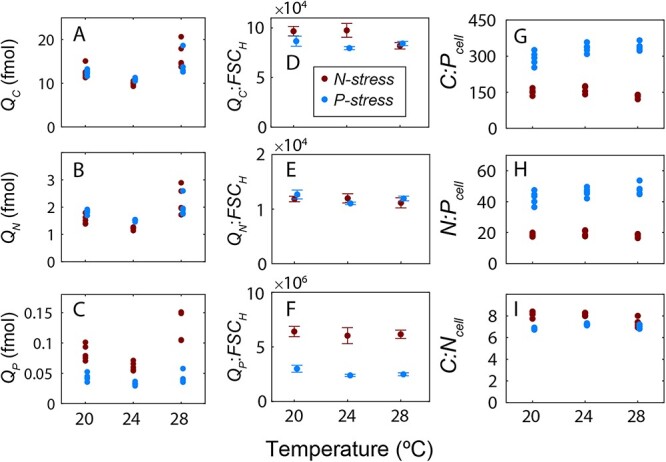
Relative influence of nutrient supply and temperature on cellular elemental quotas and ratios of marine *Synechococcus* (WH8102); (A) carbon cell quota (fmol cell^−1^), (B) nitrogen cell quota (fmol cell^−1^), (C) phosphorus cell quota (fmol cell^−1^), (D–F) means with standard deviations (*n* = 5) of cell quotas normalized to size proxy (forward scatter, *FSC_H_*), (G) C:P, (H) N:P, and (I) C:N cellular elemental ratios (mol/mol); cultures were grown at 20, 24, and 28°C and diluted at 0.18 day^−1^ with a nitrate:phosphate input ratio of 80 (P-stressed) and 1.7 (N-stressed); data between nutrient treatments are slightly offset to show data; regardless of nutrient status, *FSC_H_*, *Q_C_*, and *Q_N_* were highest at 28°C (*P* < .05, two-way ANOVA), supported by the positive effect of temperature on the cell shape determining protein MreB (Fig. S4; Tables S4 and S5); under N-stress, *FSC_H_*, *Q_C_*, and *Q_P_* were lowest at 24°C relative to other temperature treatments (*P* < .05, two-way ANOVA), supporting 24°C as *T_opt_* for element use; nutrients and temperature both had significant effects on *Q_P_:FSC_H_* (*P* < .05, two-way ANOVA), and the temperature effect was driven mostly by the difference between P-stressed cells at 20°C relative to *T_opt_* (Benjamini–Hochberg, *P* < .05), which resulted in a positive temperature effect on *C:P_cell_* under P-stress between 20°C and 28°C (Benjamini–Hochberg, *P* < .05); nutrients and temperature significantly interact to influence *C:P_cell_*, *C:N_cell_*, and *Q_C_:FSC_H_* (*P* < .05, two-way ANOVA; Table S4).

Cell size was smallest at 24°C and largest at 28°C (Fig. S4E), and changes in elemental quotas were linked to *FSC_H_*, reducing variability in *Q:FSC_H_* (Fig. 1D–F, Figure S4E, Table S4). Element-use efficiency for growth (i.e. the material needed to achieve a given cell replication rate) peaked at 24°C, thereby defining the optimal temperature (*T_opt_*) for element-use. Although mean *Q_N_* was slightly elevated under P-stress at 20–24°C, *Q_N_:FSC_H_* was relatively invariable across treatments (Fig. 1E, Table S4E), indicating that elevated N quotas under P-stress resulted from larger cells rather than increased N-density. However, we observed two deviations from the elemental quotas vs. cell size coupling. First, *Q_C_*:*FSC_H_* was elevated at low to mid temperature in N-stressed cells indicating that cells were more carbon-dense relative to other treatments (Fig. 1D). Second, *Q_P_:FSC_H_* was nearly 3-fold higher under N- vs. P-stress and slightly higher at low temperature relative to *T_opt_*, but only under P-stress (Fig. 1F). Thus, cell size and *Q* are key links to understand environmental regulation of cellular elements.

**Figure 2:**
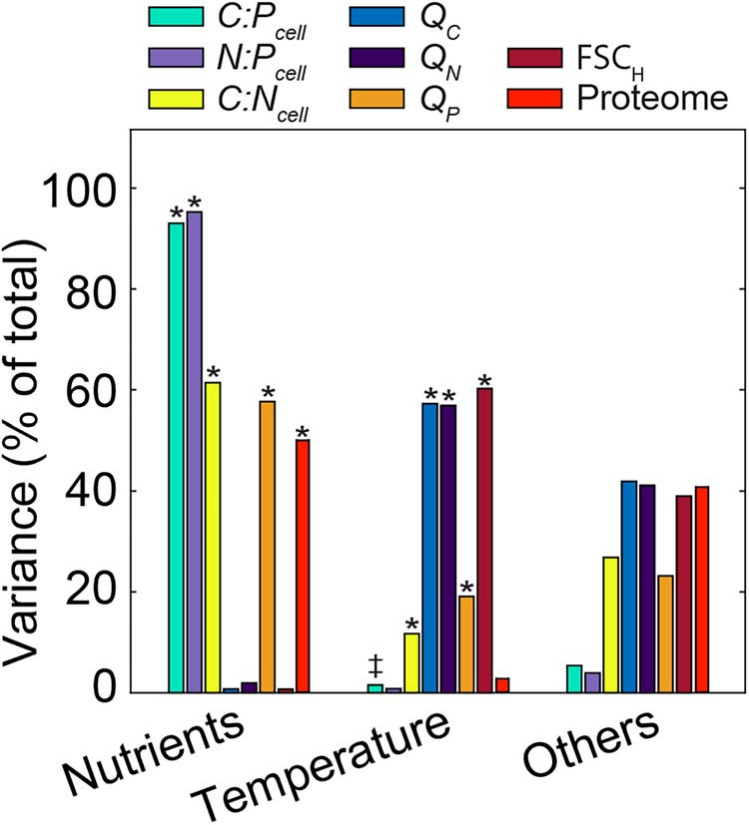
Environmental drivers of cellular quotas, ratios and proteome; portion of whole model variance of cellular elemental ratios, quotas, *FSC_H_* (two-way analysis of variance), and exclusive peak areas of all 1146 proteins (two-way permutational multivariate analysis of variance) attributable to *N:P_input_*, temperature, or other effects (includes residuals and interactive effects, ^*^ denotes environmental factor has a significant influence on relative abundance, *P* < .05); see Tables S4 and S6 for more statistical information.

We identified a clear hierarchical environmental effect on cellular elemental ratios. *N:P_input_* accounted for 93% and 95% of total *C:P_cell_* and *N:P_cell_* variances, respectively (Fig. 2), and *C:P_cell_* and *N:P_cell_* more than doubled when shifting from N- to P-stress (Fig. 1G and H). Nutrient stress effects on *C:P_cell_* and *N:P_cell_* were driven by cellular P-savings (e.g. 36% reduction of *Q_P_* under P-stress at 24°C, Table S4). Nutrient stress also impacted *C:N_cell_* (61% of variance), but the effect size was smaller (Fig. 2). *C:N_cell_* was only 5%–19% higher under N-relative to P-stress (Fig. 1I), linked to differences in *Q_C_* rather than N-density (Fig. 1D and E). Temperature explained less variance overall with 1%–2% for *C:P_cell_* or *N:P_cell_* and 10% for *C:N_cell_* (Fig. 2). However, corroborating the translation compensation hypothesis, temperature positively affected *C:P_cell_* under P-stress but not N-stress, resulting in a 17% increase between 20°C and 28°C (Fig. 1G). This suggested nutrient stress and temperature interact to influence *C:P_cell_*. Likewise, nutrient stress and temperature also interacted on *C:N_cell_*, where the *N:P_input_* effect decreased with rising temperature (Fig. 1I). In summary, nutrient stress had a primary and temperature a secondary effect on cellular elemental stoichiometry of WH8102 within the ranges of our design.

### Changes in protein-based traits

**Figure 3:**
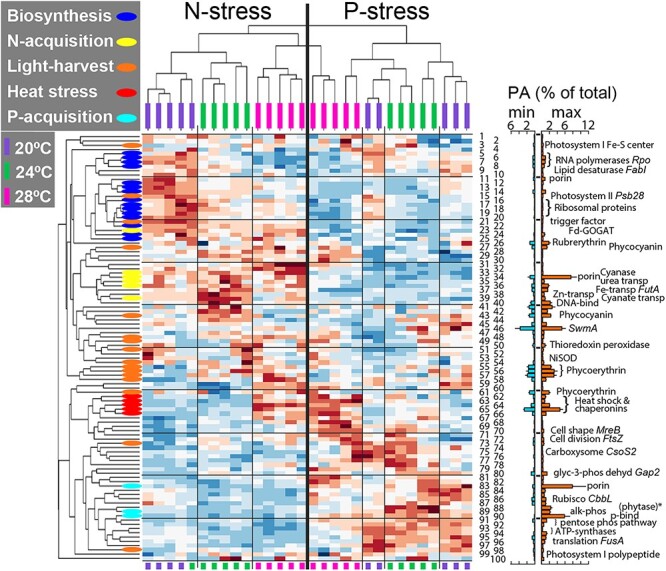
Consistent environmental response of abundant proteins; clustergram representing normalized peak areas (*PA*s) of the 100 most-abundant proteins in five replicate samples for each treatment; the clustergram function in MATLAB uses Euclidean distances in rows, correlation distances in columns, and means as linkages; the sum of mean *PA* of the 100 proteins with highest *PA* (averaged across treatments) represents ~74 ± SE 2.5% of the cumulative sum of *PA* of all proteins measured in our analysis (% *PA_Total_*; see text for explanation and Tables S5, S7, and S8 for more % *PA_Total_* detail); names of proteins in clustergram along with treatment means of % *PA_Total_* are listed in Table S7; bar chart indicates the observed minimum and maximum *% PA_Total_* means with standard deviations for the 100 most-abundant proteins; ^*^BlastP matches conserved hypothetical protein 49% with a phytase-like domain in a protein from a *Cyanobium* strain (subfamily: Synechococcoideae) and ~48% with calcium binding proteins from two other bacteria.

**Figure 4:**
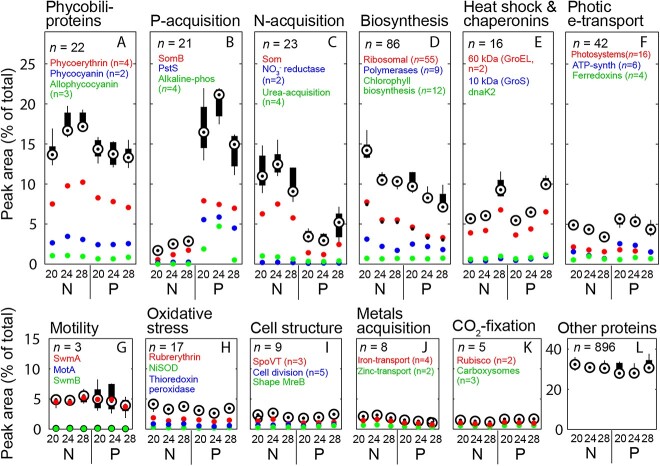
Environmental regulation of key stoichiometric traits; the percent contribution of different protein-based cell traits to the peak area (*PA*) of the whole observable proteome (% *PA_Total_*) in six steady-state continuous cultures of oceanic *Synechococcus* (WH8102) under a range of temperature (20°C, 24°C, and 28°C); either N- or P-stress is indicated with N or P, respectively (*N:P_input_* = 1.7, N; *N:P_input_* = 80, P); the sum of % *PA_Total_* was calculated for each protein group within each sample; boxplot represents the median of sums from five treatment replicate samples; boxes indicate the 25th and 75th quartiles; whiskers extend to the most extreme value that is not an outlier; outliers (non-existent in these plots) are data >1.5 times the interquartile range above or below the box; data for subgroups are means of the sum of % *PA_Total_* for all proteins within the subgroup; symbols nearest the ribosomal protein subgroup in panel D represent ribosomal proteins that likely contain rRNA and phosphorus (n = 45), denoted as ribosome group 2 in supplementary material; nutrients had the largest effects on nutrient-acquisition proteins and ribosomal proteins; temperature had the largest effects on heat shock proteins, ribosomal proteins, and proteins involved in managing photosynthetic energy flow; see Tables S6–S8 for more statistical information.

Key cellular traits varied significantly with nutrient and temperature stress. The 1146 proteins in our analysis represent >99% of the total peak area (*PA*) of the 1425 proteins that we detected (Table S2), which includes 57% of the 2512 protein-coding genes in WH8102. A two-way PERMANOVA indicated that *N:P_input_* accounted for 54% of the proteome variance (Fig. 2, Table S6). Nutrient-acquisition proteins (*NAP*s) formed the most frequent trait and responded strongly to changes in *N:P_input_* (Figs 3 and 4). Under P-stress, P-acquisition proteins including the possible porin (SomB, Q7U448), phosphate-binding protein (PstS, Q7U7G6), and alkaline phosphatases (*n* = 4, including two phytase-like proteins identified in P-blast, Q7U9T8 and Q7U862) had the largest % *PA_Total_* (Figs 3 and 4). When treatment means of % *PA_Total_* were averaged over temperature treatments (as in Table S7), allocation to P-acquisition constituted between 14% and 20% of the total proteome under P-stress (Fig. 4B; Fig. S5; Table S7). Under N stress, N-acquisition proteins were also induced but did not require the same high protein investment as P-acquisition (Fig. 4B–C). The induced N-acquisition proteins included another possible porin (Som, Q7U447) along with nitrate, nitrite, cyanate, and urea assimilation proteins. Iron and zinc acquisition proteins were also more frequent under N-stress suggesting an increased demand for metal co-factors for nitrate reduction and other N-acquisition mechanisms (Fig. 4J). When summed, all *NAP*s (P-acquisition, N-acquisition and metal transport) represented 5%–7% more of the total proteome under P-stress relative to N-stress (Fig. 4B and C and J; Table S7), thereby accounting for a portion of the elevated *Q_N_* under P-stress. Ribosomal proteins ranged from 3.3% to 7.7% of *PA_Total_* and the nutrient-wise effect from the 2-way ANOVA on this group was large (Fig. 4D; Tables S7 and S8). Within temperature treatments, relative ribosomal protein abundances were 37%–40% lower under P- vs. N-stress, with reduced contributions to *PA_Total_* by 2%–3% Fig. 4D (Fig. 4D; Tables S7 and S8). Thus, P-stress resulted in the largest increase in a single trait (all *NAPs*, Fig. S4F) and the largest decrease in P-rich ribosomal proteins. To illustrate this influence on *C:P_cell_* stoichiometry, we compared the ratio of *NAP* to calculated estimates of rRNA and identified a major correspondence between nutrient-wise changes in *C:P_cell_* and ratios of *NAP:rRNA* investments (Fig. S4H). Overall, the proteome responded dynamically, with *NAP*s and ribosomal proteins representing the strongest responses to nutrient stress.

Temperature had an additional albeit weaker influence on the proteome and accounted for 2.8% of the variance (Fig. 2). The heat-stress proteins, dominated by the chaperonins and co-chaperonins (GroES (Q7TTX2), GroELS (Q7TTX1, Q7TTT6), DnaK2 (Q7U3C4)), increased in relative abundance from 20°C to 28°C. As a result, the heat stress trait comprised ~10% of the total proteome at 28°C (Fig. 4E, Table S7). This heat stress trait responded orthogonally with the biosynthesis trait. As temperature increased from 20°C to 24°C, relative ribosomal protein abundances declined by 29% under N-stress and 24% under P-stress (Fig. 4D, Table S7 and S8). Only minor declines were seen when shifting to 28°C. Similarly, protein allocations for photosynthetic electron transport and ATP synthesis also decreased with increasing temperature suggesting a wider thermal effect on core metabolic functions (Fig. 4D, Tables S7 and S8). Furthermore, our analysis identified interactive relationships between temperature and nutrients on multiple biochemical functions. These functions included ATPases, phycobiliproteins, the oxidative pentose phosphate (PP) pathway, and enzymes involved in cell structure (Fig. 4; Fig. S6; Tables S6–S8). Although N-stress and temperature had a positive interactive effect on relative abundances of phycobiliproteins (Fig. 4A; Tables S6–S8), P-stress and temperature had a positive interactive effect on relative abundances of glucose-6-phosphate dehydrogenase and OpcA (Q7U875) that support the oxidative PP pathway (Fig. S6) [39, 40]. The oxidative PP pathway supplies NADPH, a process commonly associated with the dark cycle in Cyanobacteria [41], but here, was favored in the middle of the light period under P-stress relative to N-stress. Overall, thermal influences included a robust positive effect on heat-shock proteins across nutrient treatments, compensatory responses with declining temperature, and interactive effects with nutrients on processes involved in relative carbon accumulation and use.

### Changes in central metabolism

To explore how shifts in central metabolism influence cellular elemental allocations, we applied a NoB-LoC algorithm. Our analysis high-lighted shifting carbon metabolism, compounds involved in osmotic regulation, cell wall biosynthesis, and poly-P accumulation as additional traits affecting cell quotas. First, several proteins involved in glycogen utilization were more abundant under P- relative to N-stress (Fig. S6). Although the glycogen synthesis enzyme, 1,4-alpha-glucan branching enzyme (GlgB, Q7U646, Bicluster 4) was only slightly induced under N-stress relative to P-stress at 20°C, other glycogen synthesis enzymes including glycogen synthase (GlgA, Q7U7I2, Bicluster 7) and glucose-1-phosphate adenylyltransferase (GlgC, Q7U768, Bicluster 5) were stable between temperature treatments (Fig. S6), indicative of weak support for upregulated glycogen synthesis pathways under N-stress. Instead, the glycogen digestive enzyme α-1-4 glucan phosphorylase was more frequent under P-stress and glycosyl hydrolase (Q7U4W1, Bicluster 8), a versatile enzyme class that may also be involved with sugar degradation, also clustered with several established P-stress proteins regardless of temperature (Table S3). Combined, relative changes in abundances of these digestive enzymes suggested elevated organic carbon use and therefore less accumulation under P-stress (Fig. S6). Although we did not measure glycogen concentrations directly, the results align well with the reduced *Q_C_*:*FSC_H_* under P-stress. Second, expression of glucosyl-3-phosphoglycerate synthase (Q7U3J6) clustered with relative abundances of several proteins clearly involved in N-stress (Table S3, Bicluster 5). Glucosyl-3-phosphoglycerate synthase supports replacement of glutamate with the N-free, C-rich compatible solute glycosyl-glycerate (GGA) under N-stress in *Synechococcus* [42, 43]. This result aligns with the increased *Q_C_*:*FSC_H_* under N-stress and comparative calculations of *C:N_cell_* with GGA replacement align with observed *C:N_cell_* measurements (Table S11). Third, we observed a putative P-stress-dependent regulation of precursors to peptidoglycan and lipopolysaccharides (LPSs). These pathways including N-acetyl-glucosamine-6-phosphate deacetylase (NagA, Q7U3Z1, Bicluster 8), N-acetylmuramic acid 6-phosphate etherase (MurQ, Q7U6S0, Bicluster 3), and the bifunctional protein for UDP-N-acetylglucosamine (GlmU, Q7U7I0, Bicluster 3) [44, 45] are involved with metabolism of either cell wall or membrane components and clustered with several P-acquisition proteins (replotted in Fig. S6; Table S3). Collectively, this suggests that the biosynthesis pathway to UDP-N-acetylglucosamine and the placement of this monomer in either peptidoglycan or LPS is more active under P-stress relative to N-stress. Elevated cell concentrations of peptidoglycans and N-enriched, cross-linked oligopeptides under P-stress align with elevated N-quotas. Fourth, our calculations indicate that temperature had a positive influence on the portion of *Q_P_* that is apportioned to cell components other than nucleic acids (Fig. S4B) and on enzymes controlling the synthesis vs. degradation of polyphosphate (Fig. S4C and D). In sum, we observed nutrient- and temperature-stress effects on key metabolic pathways that are involved in cellular use of carbon, nitrogen, and phosphorus.

## Discussion

### Existing hypothesis for biochemical regulation of *C:N:P_cell_*

We found mixed support for existing hypotheses describing elemental allocation in *Synechococcus* [11]. Elemental quotas, ratios, and *FSC_H_* at 24°C aligned with previous data from chemostat cultures of WH8102 [29] and supported the nutrient supply hypothesis for *C:P_cell_* and *N:P_cell_*, mostly through differences in the P-quota. However, *N:P_input_* interacted with temperature to affect *C:N:P_cell_* through the thermal influence on ribosomes that may have arisen from the translation compensation mechanism. We interpret this interactive environmental effect as driven by high P-quotas under N-stress, which overwhelms a small thermal effect on ribosomes and associated P-requirements. This interpretation is partially supported by the large *N:P_input_* effect on ribosomal proteins, which contributes to the nutrient-wise effect on *Q_P_:FSC_H_*. Thus, ribosomes add to a list of biochemicals, such as phospholipids [46], phosphorylated phycobiliproteins [47], polyphosphates [16], and P-storage [15] that can harbor P under P-replete conditions. To estimate allocations among P-pools, we rely on other data [25] to calculate that P-savings from sulfolipid replacement in WH8102 only reduced *Q_P_* by 2%, similar to measured estimates [46]. However, rRNA and unidentified pools reduced *Q_P_* by 10% and 24%, respectively, at 24°C. In support of a previous hypotheses regarding nutrient-acquisition proteins [21, 48], P-acquisition proteins along with N in peptidoglycans can account for a portion of the increase in *Q_N_* and *FSC_H_* under P-stress. As *Q_C_* and *Q_N_* are linked through proteins and peptidoglycans, high relative abundances of these integral membrane/wall structures may be important traits that contribute to cell size and elemental ratios. In sum, the translation compensation mechanism may have impacted *Q_P_:FSC_H_* and *C:P_cell_* under P-stress but not N-stress due to the overwhelming *N:P_input_* effect on *Q_P_:FSC_H_*. The *N:P_input_* effect was also large but opposite in sign on *NAP*. These opposing effects on ribosomes and *NAP* combined to amplify nutrient-wise differences in *C:P_cell_* and *N:P_cell_*. Because chemostat dilution rates are similar to implied rates in ocean gyres [49], our interpretations are likely applicable to field data.

### New and alternate hypotheses for biochemical regulation of *C:N:P_cell_*

Our proteomics analysis allowed for new perspectives of biochemical regulation of *C:N:P_cell_*. Nutrient regulation of *C:N_cell_* was not strong but the interactive response with temperature requires careful analysis. As hypothesized from Droop-like models [19], we detected higher *C:N_cell_* under N-stress relative to P-stress, but not at high temperature. In contrast to other data that identify large variability in *Q_N_* as a function of *N:P_input_* [46, 50], *Q_N_:FSC_H_* was nearly constant between treatments. Instead, changes in *C:N_cell_* were driven by *Q_C_:FSC_H_*. Multiple studies have identified broad correspondence between cell volume and carbon biomass but have also identified considerable variability within a size class and associated variation in cellular carbon density [51, 52]. Our data suggest at least two pathways for size-independent increases in cellular carbon density under N- compared to P-stress. First, P-stress induced multiple pathways for carbon respiration, whereas N-stress induced only minor support for elevated glycogen production. Second, N-stress supported high glucosyl-3-phosphoglycerate synthase abundance, the enzyme responsible for replacing N-rich glutamate with the C-rich compatible solute GGA [43], and our hypothetical calculations of *C:N_cell_* with GGA replacement support previous data regarding GGA in Cyanobacteria [43, 53]. Third, the interactive treatment effect on *C:N_cell_* may be due to thermally-driven increases in N-rich phycoerythrin abundances under N-stress. This result here is different than other observations of degraded phycobiliproteins under N-stress [22] but has been observed in a mutant strain of *Synechococcus* devoid of a glycogen synthesis enzyme [54], a condition similar to the weak support for an N-stressed glycogen synthesis process that we observed in WH8102. Slow-growing, acclimated, N-stressed cells may require high phycoerythrin abundances to manage energy flow in the absence of an efficient carbon-overflow mechanism, perhaps through state transitions [55]. Overall, our proteomic results suggest a more complex regulation of *C:N_cell_* in marine phytoplankton than previously recognized.

Although we identified biochemical support for the translation compensation hypothesis, the temperature effect on *Q_P_:FSC_H_* was small between 20°C and 28°C and other hypotheses may be more important for *Q_P_* dynamics and marine ecosystems within this thermal range. As hypothesized, we observed a negative relationship between temperature and ribosomal proteins [11, 17]. However, the thermal effect on ribosomal proteins and *C:P_cell_* only seems observable under P-stress, when other P-resources, like polyphosphates or periplasm-P are minimized or depleted. By comparison, the nutrient-wise effect on ribosomal proteins was large, a trend supported in previous studies of *Synechococcus* WH8102 [29, 56]. Elevated ribosome abundances under P-repletion may scavenge P at an N-cost in non-active ribosomes [55]. Alternatively, streamlined efficiency [57] of ribosomes under P-stress could result from high production of abundant proteins like PstS and alkaline phosphatases. In either case, P-supply has opposing effects on *Q_CN_* and *Q_P_* through *NAP* and ribosomes, respectively, that together contribute to large changes in *C:P_cell_* and *N:P_cell_*. Because this efficiency ratio of *NAP*:ribosomes peaked at 24°C (supporting other estimates of *T_opt_* for WH8102 [58]), along with cell carrying capacity (Fig. S1A), and elevated protein chaperone abundances suggest thermal stress at 28°C [33, 59, 60], this efficiency mechanism may be important for *Synechococcus* ecology. For example, the cell-shape-determining protein MreB [61] or cell division metrics [62] may be important regulators of microdiversity because of inherent links between cell size, *T_opt_* for element-use efficiency and carrying capacity.

There are caveats for linking our experiments with large-scale regulation of C:N:P. First, our investigation using DIA-MS proteomics approaches a comprehensive analysis but future investigations of biodiversity in cellular P dynamics will help to delineate *Q_P_* regulation. Second, our analysis of % *PA_Total_* approximates relative protein investments into specific traits rather than relative comparisons of protein copy numbers between treatments. Third, due to the complexity of chemostat experiments, we only examined a single strain under limited environmental conditions, whereas variability in the field includes broader conditions and more diverse phytoplankton lineages. For example, % P-savings from sulfolipid replacement are variable between strains of *Synechococcus* [25]. Fourth, our definition of *T_opt_* for element-use efficiency is different than the definition of *T_opt_* for growth rate and seems more relevant under nutrient limitation. Fifth, our proteome analysis excludes proteins that are not well-represented or absent across treatments. Despite these caveats, our molecular information helps constrain the regulation of phytoplankton biochemistry. Exploring more lineages, environmental conditions, and biochemical assays will improve our understanding ocean C:N:P.

### Implications for field observations

Field observations indicate that nutrient stress drives C:N:P in low-latitude ecosystems, where the thermal effect is relatively small [9]. Similarly, temperature had little effect on *C:P_cell_* and *N:P_cell_* in our cultures under N stress—the most frequent nutritional condition observed across oceans [63]. However, ecosystem observations do indicate that C:P and N:P are slightly depressed at high temperature, possibly due to rapid growth [49] or heat-stress [9]. Conversely, in high-latitude, cold ecosystems, temperature shifts play a stronger role in driving C:N:P variability compared to the thermal range in our design [9, 17]. The relatively weak influence of temperature on *C:N:P_cell_* observed here implies that lineage-wise variability in C:N:P or thermal influences in other lineages are stronger in the field. Thus, shifts in biodiversity may contribute to C:N:P variability in the surface ocean beyond the physiological mechanisms described here. Hence, the combined field and experimental data suggest complex effects on C:N:P in marine ecosystems that incorporate current hypotheses and evolving theories.

## Supplementary Material

ISMEJ-D-23-00130R2_suppl_figures_(clean)_wrae046

TS1_Growth_medium_recipe_wrae046

TS2_Peak_area_dataset_comparisons_wrae046

TS3_Biclusters_wrae046

TS4_Elemental_quotas_and_ratios_wrae046

TS5_Individual_protein_statistics_and_PA_wrae046

TS6_Permanova_protein_groups_wrae046

TS7_Protein_groups_names_treatment_means2_wrae046

TS8_Protein_groups_statistics2_wrae046

TS9_Normalized_excl_PA_1438_wrae046

TS10_Normalized_excl_PA_1236_wrae046

TS11_CN_ratio_with_GGA_wrae046

## Data Availability

Cell data are accessible in Table S4. Proteomic data are available in Tables S9 and S10, via ProteomeXchange (PXD043180), and at https://www.bco-dmo.org/dataset/923159 [64]. Codes and instructions for implementing the Nonparametric Bayesian Local Clustering algorithm on proteomics are available on GitHub at https://github.com/mingyudu/NoB-LoC.
