# Supplementary material for: Proteome trait regulation of marine Synechococcus elemental stoichiometry under global change
Source: ISME J. 2024 Mar 21;18(1):wrae046. doi: 10.1093/ismejo/wrae046 (PMC11020310; doi:10.1093/ismejo/wrae046)
Supplement: ISMEJ-D-23-00130R2_suppl_figures_(clean)_wrae046 [file ismej-d-23-00130r2_suppl_figures_(clean)_wrae046.docx]

**Supplementary Figures**


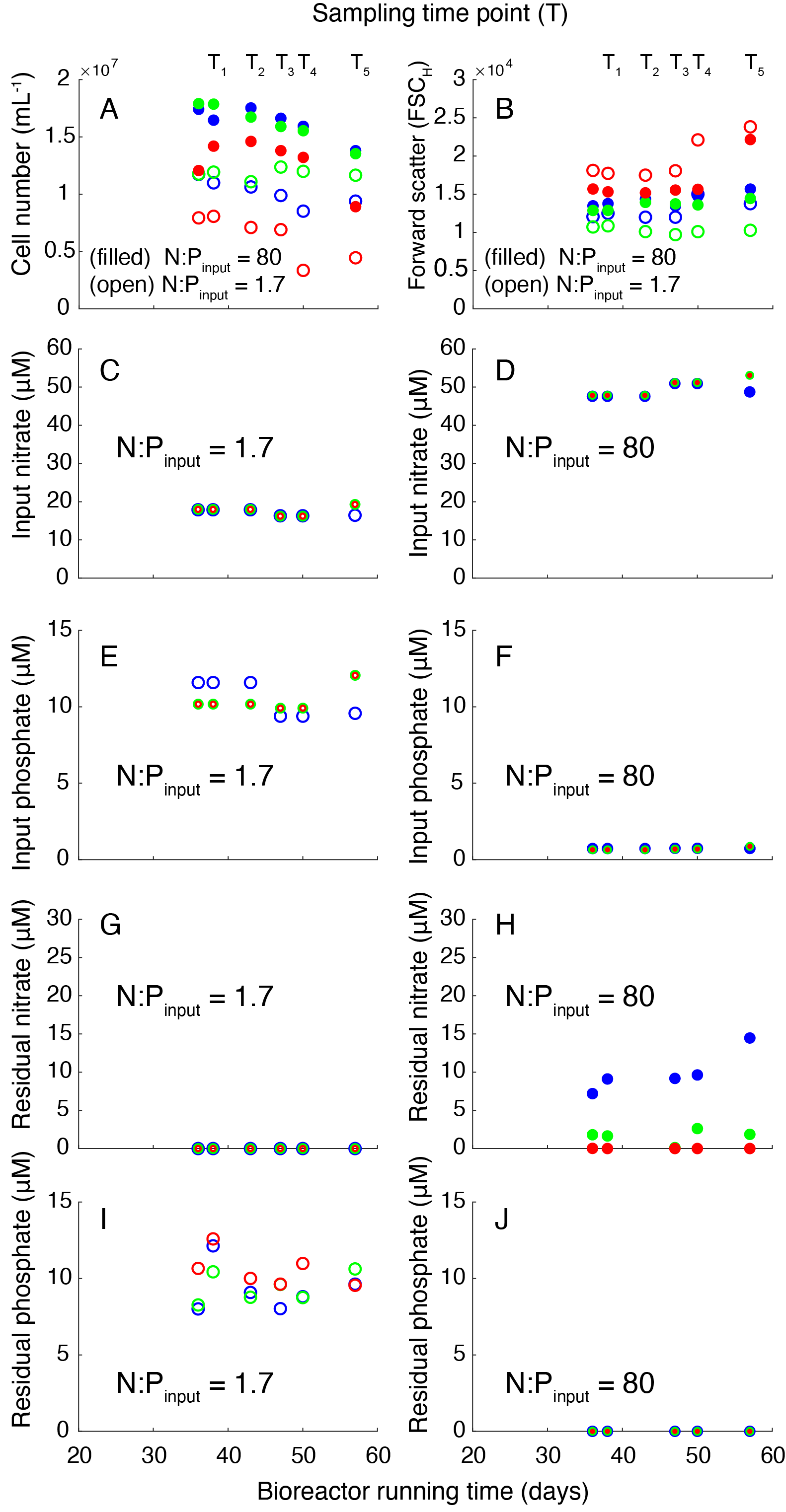


**Supplementary Figure S1. Cell density (A) and cell size (FSC_H_; B) in chemostat cultures of WH8102 over time (days) since chemostat initiation.** Input concentrations of nitrate (C, D) and phosphate (E, F) in media with *N:P_input_* ratios of 1.7 (C, E) and 80 (D, F). Residual nitrate (G, H) and phosphate (I, J) in chemostat reservoirs with *N:P_input_* ratios of 1.7 (C, D) and 80 (E, F). Blue, green, and red symbols represent 20°C, 24°C, and 28°C, respectively. Sampling time points (T_1-5_) are indicated above plots.


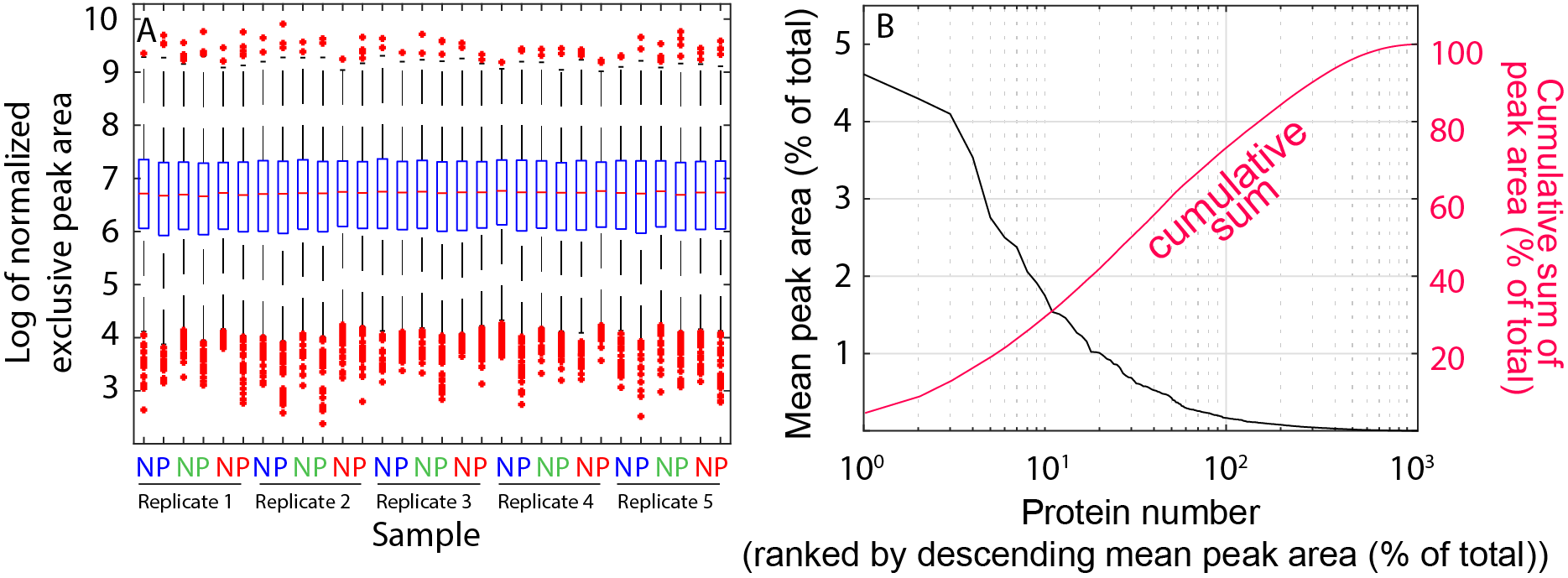


**Supplementary Figure S2. Peptide peak area normalization and contributions to the total peak area of the observable proteome.** A) Log of normalized exclusive peak area (*PA*) values of all observable proteins identified with 2 or more peptides normalized with the Scaffold DIA Proteome Software (Scaffold Proteome Software, Inc.). Nutrient stressor is indicated by N or P and color code blue, green, and red indicate temperature treatments of 20°C, 24°C, and 28°C. B) The mean % *PA_Total_* of individual proteins across samples is ordered by descending % *PA_Total_* and is plotted against the left y-axis. Against the right y-axis is the cumulative sum of the % *PA_Total_*. The % *PA_Total_* calculation is explained in the text.


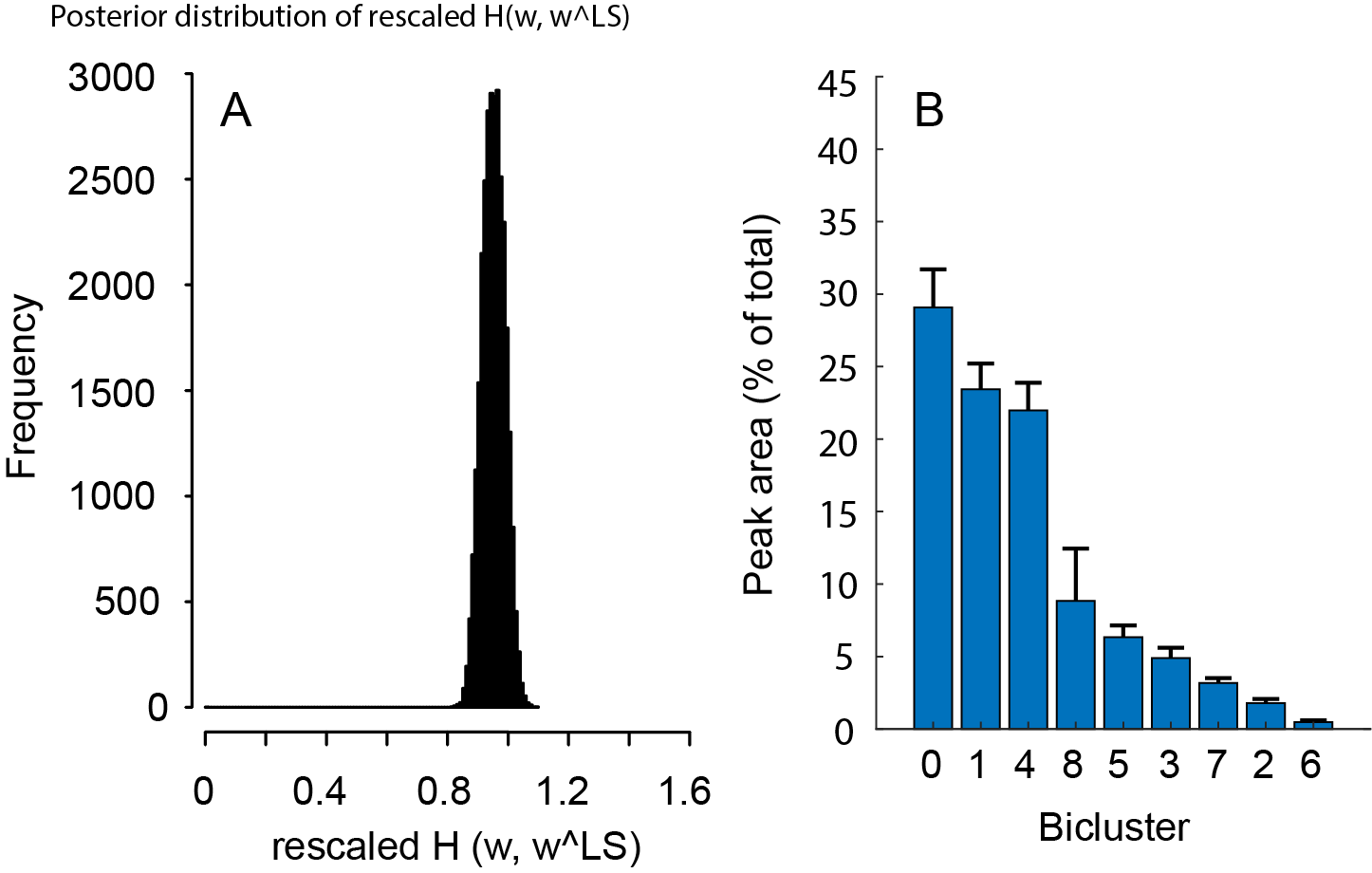


**Supplementary Figure S3. Results from the Nonparametric Bayesian Local Clustering algorithm. A**) Posterior distribution of the scaled distance metric for the parameter $\omega$. B) Bicluster representation of total proteome - rank of sum of mean % peak areas with standard deviation across 30 samples for all proteins included in biclusters.


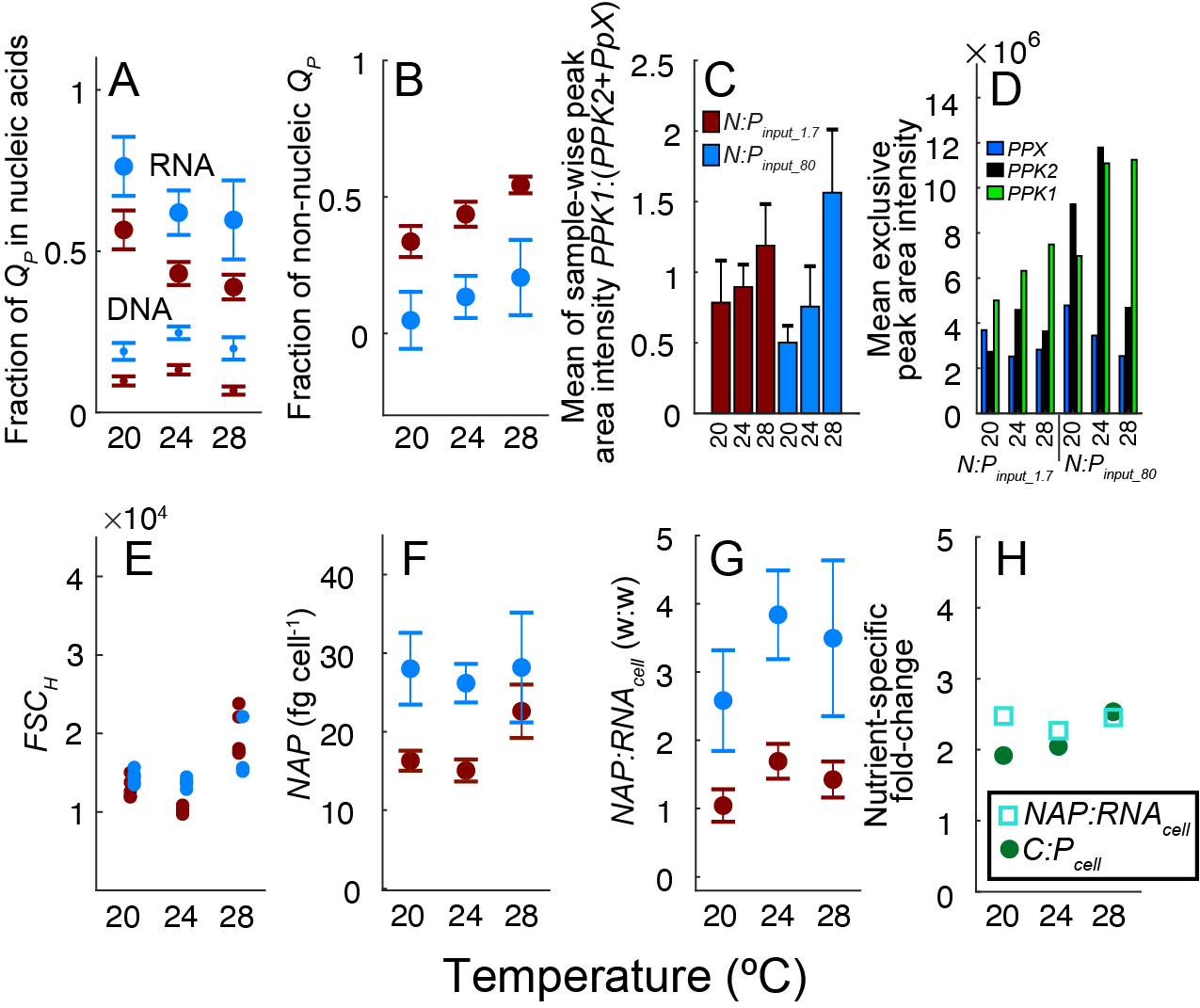


**Supplementary Figure S4. Calculated distribution of P among cellular resources indicates that the nutrient treatment effect size on *C:P_cell_* and nutrient acquisition proteins *(NAP):RNA_cell_* are similar.** To begin this analysis, we first calculated the fraction of *Q_P_* in RNA and DNA, which serves as a reference. The weight of RNA was calculated based on a calculated estimate of protein weight (based on *Q_N_*) and the relative ribosomal protein percentage from the % *PA_Total_* calculations. To do this we 1) the calculated cellular mass of proteins using a *Q_N_*:cellular protein (w:w) conversion factor of 5.0[1, 2], 2) the % *PA_Total_* of ribosomal proteins that contain RNA (denoted as ribosomal protein group 2 in Table S7), 3) an *RNA:protein_ribosome group_2_* (w:w) ratio of 2[3], 4) the weight of one copy of a WH8102 genome using a conversion factor of 1base pair = 650 Daltons and 5) a weight-based conversion factor for P in RNA (0.091) and for DNA (0.095). We then compared the nutrient-specific change in the means (n=5) of *NAP:RNA_cell_* against that in *C:P_cell_*. A) Fraction of *Q_P_* in *RNA_cell_* (large symbols) and *DNA_cell_* (small symbols) with standard deviations. Blue symbols are P-stressed (*N:P_input_* = 80) and dark red symbols are N-stressed (*N:P_input_* = 1.7). B) Fraction of *Q_P_* in resources other than nucleic acids. C) Treatment means and standard deviations of ratios of peak areas of anabolic and catabolic polyphosphate enzymes. Ratios increased significantly with temperature, suggesting polyphosphate production rates increased with temperature (2-way ANOVA, *p*<0.001). D) Treatment group means of exclusive peak areas of enzymes involved in polyphosphate synthesis (Ppk1) and catabolism (Ppx and Ppk2; identifying references[4, 5]. E) Forward scatter (*FSC_H_*) of particles in chemostat cultures of *Synechococcus* WH8102 across temperature and nutrient treatments at 5 sampling points. Data between nutrient treatments are offset on plots to show data points. F) Total calculated abundances of nutrient acquisition proteins (*NAP*) includes N-, P- and metals-acquisition proteins G) Total calculated abundance ratios of *NAP*:*RNA* (w:w). H) Comparison of the nutrient specific fold-change in the total calculated abundance ratios of *NAP:RNA* (w:w) with the nutrient-specific fold-change in *C:P_cell_*. See Tables S4 and S5 for statistical information.


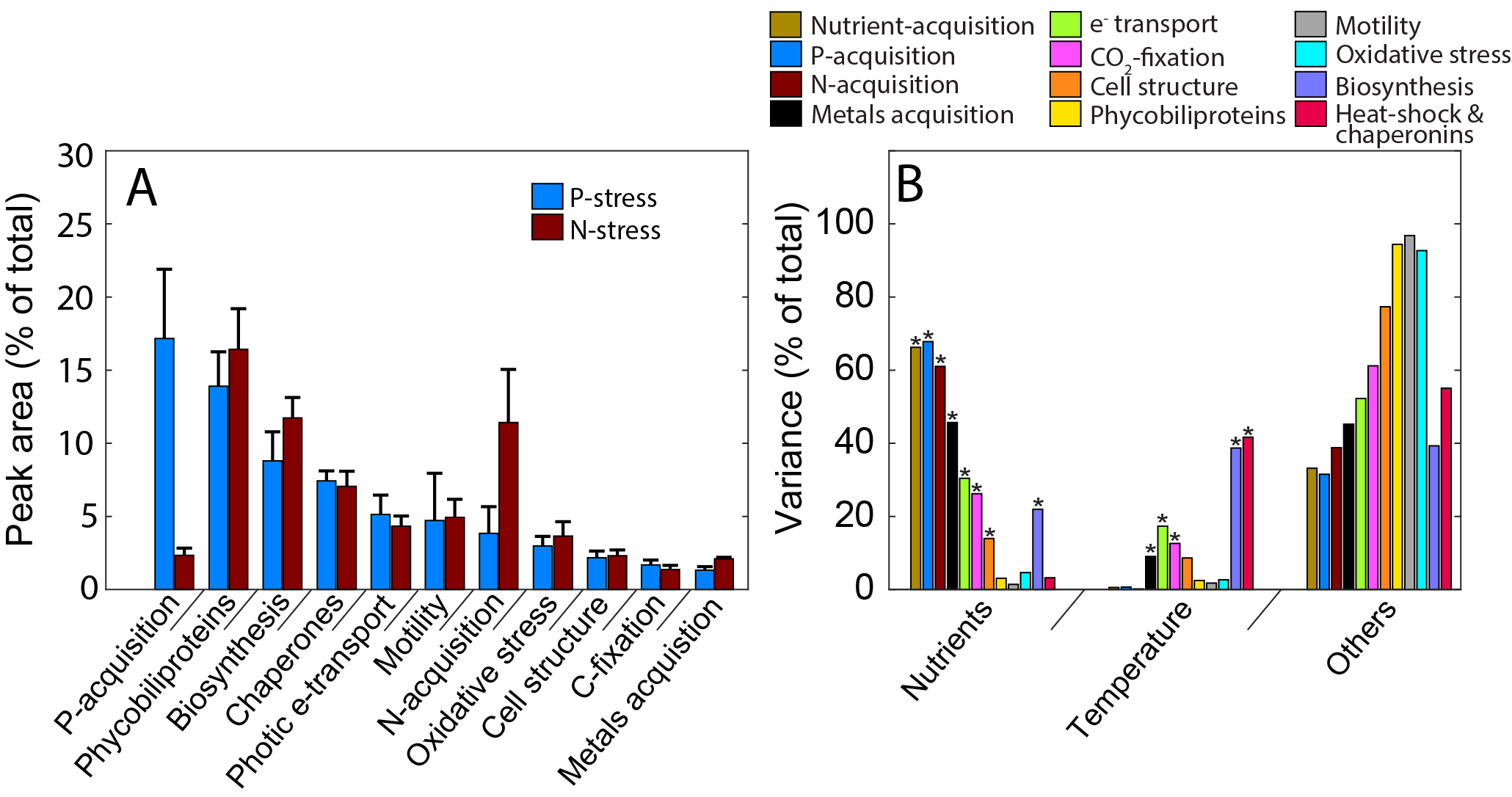


**Supplementary Figure S5. Regulation of key stoichiometry traits.** A) Rank of mean relative abundance by % peak area of the whole proteome (% *PA_Total_*) for 11 protein-based trait groups under P-stress and N-stress. Error includes the standard deviation of the mean of treatment replicates % *PA_Total_* (*n*=5) propagated onto the nutrient-group-wise mean. B) Portion of whole model variance (2-way PERMANOVA) of peak areas of 12 proteins groups attributable to *N:P_input_*, temperature and other effects (includes residuals and interactive effects) in steady-state cultures of *Synechococcus* WH8102 (*indicates *p*<0.05, df = 1). The nutrient acquisition group includes N-, P- and metals-acquisition proteins. See Supplementary Tables S6-S8 for more statistical information.


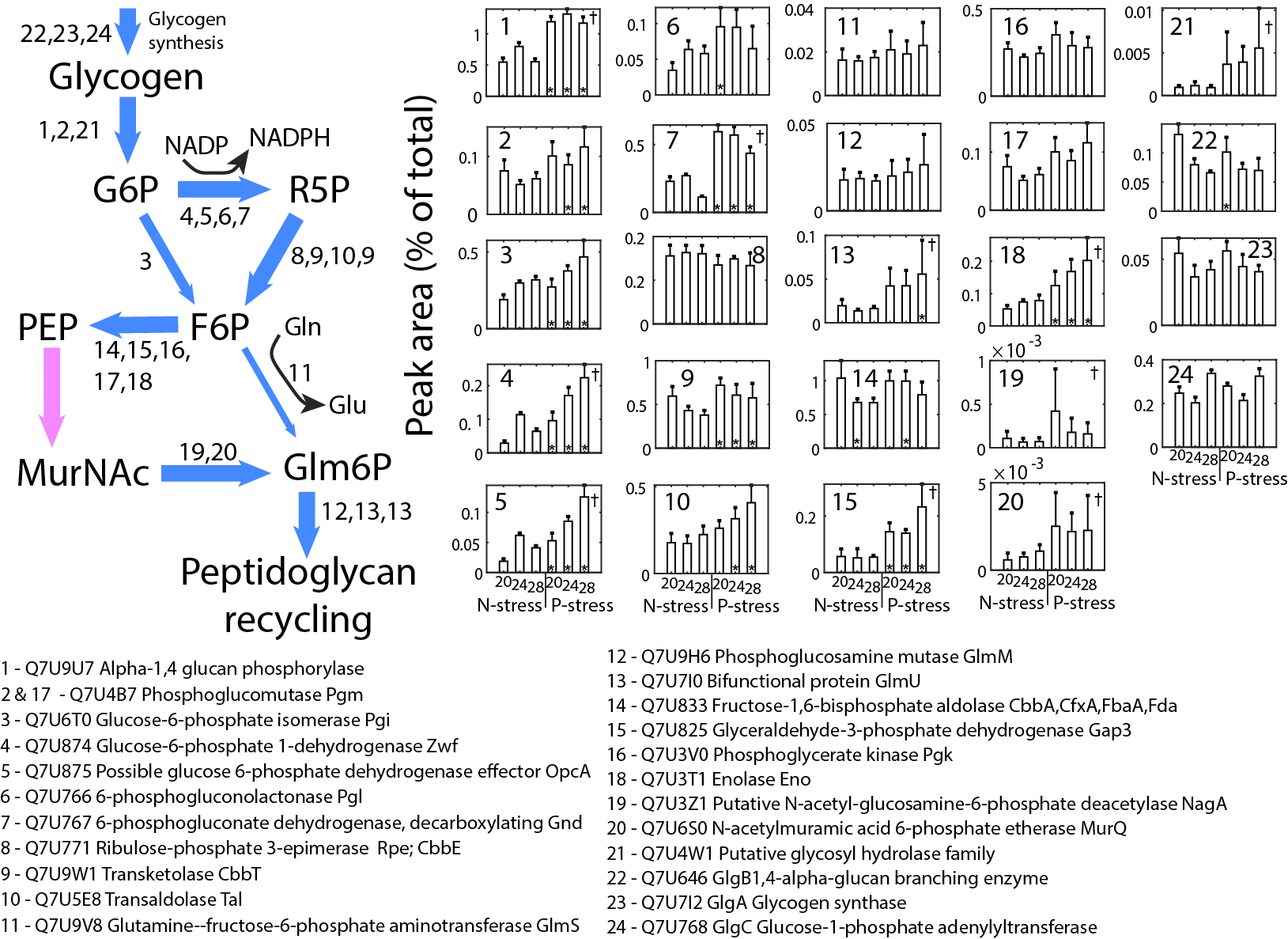


**Supplementary Figure S6. Enzymes representing glycogen metabolism, the oxidative pentose phosphate pathway and pathways to peptidoglycan recycling and lipopolysaccharide production in chemostat cultures of WH8102 across treatments of temperature (20°C, 24°C, 28°C) and nutrients (N- and P-stress).** The Nonparametric Bayesian Local Clustering algorithm indicates these pathways are supported under P-stress relative to N-stress in *Synechococcus* WH8102. Enzymes and identifiers are listed by numbers 1-24. G6P, Glucose-6-phosphate; R5P, Ribulose-5-phosphate; F6P, Fructose-6-phosphate; PEP, Phosphoenol pyruvate; MurNAc, N-acetyl-muramic acid; Glm6P, Glucosamine-6-phosphate; Gln, glutamine; Glu, glutamate. PEP is the putative phosphorylating mechanism of the phosphate transport system for MurNAc. Pathways are supported in literature[6–8]. *Indicates % *PA_Total_* under P-stress is different than under N-stress at the same temperature (*p*<0.05, Benjamini-Hochberg). † Clusters with P-acquisition proteins in the non-parametric Bayesian local clustering analysis. See Supplementary Figure S3 for more information on the non-parametric Bayesian local clustering analysis and Supplementary Tables S3 and S5 for other statistical information.

**References**

1. Lourenço SO, Barbarino E, Marquez UML, Aidar E. Distribution of intracellular nitrogen in marine microalgae: Basis for the calculation of specific nitrogen-to-protein conversion factors. *J Phycol* 1998; **34**: 798–811.

2. Grossmann L, Hinrichs J, Weiss J, Org Hinrichs J. Cultivation and downstream processing of microalgae and cyanobacteria to generate protein-based technofunctional food ingredients. *Crit Rev Food Sci Nutr* 2019; **60**: 2961–2989.

3. Geider R, La Roche J. Redfield revisited: variability of C:N:P in marine microalgae and its biochemical basis. *Eur J Phycol* 2002; **37**: 1–17.

4. Gray M, Wholey W, Wagner N, Cremers C. Polyphosphate is a primordial chaperone. *Mol Cell* 2014; **53**: 689–699.

5. Rao NN, Gómez-García MR, Kornberg A. Inorganic polyphosphate: essential for growth and survival. *Annu Rev Biochem* 2009; **78**: 605–647.

6. Park JT, Uehara T. How bacteria consume their own exoskeletons (turnover and recycling of cell wall peptidoglycan). *Microbiol Mol Biol Rev* 2008; **72**: 211–227.

7. Dahl U, Jaeger T, Nguyen BT, Sattler JM, Mayer C. Identification of a Phosphotransferase System of *Escherichia coli* Required for Growth on N-Acetylmuramic Acid. *J Bacteriol* 2004; **186**: 2385–2392.

8. Guyet U, Nguyen NA, Doré H, Haguait J, Pittera J, Conan M, et al. Synergic Effects of Temperature and Irradiance on the Physiology of the Marine *Synechococcus* Strain WH7803. *Front Microbiol* 2020; **11**: 1707.
