# Supplementary material for: Proteome trait regulation of marine Synechococcus elemental stoichiometry under global change
Source: ISME J. 2024 Mar 21;18(1):wrae046. doi: 10.1093/ismejo/wrae046 (PMC11020310; doi:10.1093/ismejo/wrae046)
Supplement: TS11_CN_ratio_with_GGA_wrae046 [file ts11_cn_ratio_with_gga_wrae046.docx]

| Table S11. Calculations for hypothetical *C:N_cell_* with glucosyl glycerate replacement of glutamate from cellular elemental quotas (*Q_C_* and *Q_N_*) measured in the P-stress treatment. | | | | | | |
| --- | --- | --- | --- | --- | --- | --- |
| Temperature treatment | 20°C | | 24°C | | 28°C | |
| Nutrient supply treatment | N-stress | P-stress | N-stress | P-stress | N-stress | P-stress |
| Measured mean *Q_C_* (fmol) | 12.63 | 12.46 | 9.94 | 10.92 | 16.32 | 14.13 |
| Measured mean *Q_N_* (fmol) | 1.55 | 1.82 | 1.22 | 1.51 | 2.23 | 2.00 |
| Measured mean *C:N_cell_* | 8.16 | 6.84 | 8.14 | 7.21 | 7.32 | 7.06 |
| Calculated glutamate N (fmol) (4% of *Q_N_*) |  | 0.07 |  | 0.06 |  | 0.08 |
| Calculated glutamate C (fmol) (C:N_glutamate_ = 5) |  | 0.36 |  | 0.30 |  | 0.40 |
| Hypothetical *Q_C_* (fmol) without glutamate |  | 12.10 |  | 10.62 |  | 13.73 |
| Hypothetical *Q_N_* (fmol) without glutamate |  | 1.75 |  | 1.45 |  | 1.92 |
| Calculated GGA C (fmol) 9C:0N with 3-fold increase in [GGA_cell_] over original [glutamate_cell_] |  | 1.48 |  | 1.23 |  | 1.62 |
| Calculated *Q_C_* (fmol) with glucosyl glycerate replacement |  | 13.57 |  | 11.84 |  | 15.35 |
| Calculated *Q_N_* (fmol) with GGA replacement of 75% glutamate |  | 1.77 |  | 1.47 |  | 1.94 |
| Calculated mean *C:N_cell_* with GGA replacement from P-stress treatment |  | 7.68 |  | 8.06 |  | 7.91 |

To estimate a potential effect of glucosyl glycerate (GGA) replacement of glutamate on *C:N_cell_*, we rely on previous data [1] that identify a ~75% decrease in cellular glutamate concentrations (5C:1N) under N-stress and a ~3-fold increase in cellular concentrations of GGA (9C:0N) over N-replete cellular concentrations of glutamate in response to N stress. Since [glutamate_cell_] is more than 2 orders of magnitude higher than the most commonly encoded amino acid Leucine [2] we assume glutamate N is ~4% of *Q_N_* [3] if the maximum growth rate of WH8102 is similar to that for WH8103 at ~1.0 day^-1^ [4]. We then use our measured mean *C:N_cell_* values under P-stress (Table TS4) to calculate a hypothetical mean *C:N_cell_* that would exist under N-stress if GGA replaced cellular glutamate with these inputs. Our calculated hypothetical mean *C:N_cell_* values under P-stress with GGA replacement (7.68, 8.06, 7.91 at 20°C, 24°C, and 28°C, respectively) are similar to our measured mean C:N values under N-stress (8.16, 8.14, and 7.32 at 20°C, 24°C, and 28°C, respectively, Table TS4). These comparisons between our hypothetical mean C:N values and measured mean C:N values suggest that temperature may also be a negative factor in influencing cellular GGA concentrations in WH8102, which has been observed in other Cyanobacteria [5]. Alternatively, there are other possible factors that may have contributed to *C:N_cell_* measurements including those described in the main text, such as phycobiliproteins and central carbon metabolism.

References

1. Klähn S, Steglich C, Hess WR, Hagemann M. Glucosylglycerate: A secondary compatible solute common to marine Cyanobacteria from nitrogen-poor environments. *Environ Microbiol* 2010; **12**: 83–94.

2. Walker MC, Van Der Donk WA. The many roles of glutamate in metabolism. *J Ind Microbiol Biotechnol* 2016; **43**: 419–430.

3. Rhee GY. Effects of N:P atomic ratios and nitrate limitation on algal growth, cell composition, and nitrate uptake. *Limnol Oceanogr* 1978; **23**: 10–25.

4. Moore LR, Goericke R, Chisholm S. Comparative physiology of *Synechococcus* and *Prochlorococcus*: influence of light and temperature on the growth, pigments, fluorescence and absorptive properties. *Mar Ecol Prog Ser* 1995; **116**: 259–275.

5. Warr SRC, Reed RH, Stewart WDP. Carbohydrate accumulation in osmotically stressed Cyanobacteria (blue-green algae): interactions of temperature and salinity. *New Phytol* 1985; **100**: 285–292.
